# Harnessing AI in Laser Aesthetic Treatments: Revolutionizing Precision, Safety, and Personalization

**DOI:** 10.1111/jocd.16704

**Published:** 2024-11-27

**Authors:** Diala Haykal

**Affiliations:** ^1^ Private Practice Centre Laser Palaiseau Palaiseau France

## 1 Introduction

In the last decade, the aesthetic dermatology field has witnessed groundbreaking innovations, with laser technology at the forefront. Lasers have evolved to address a wide range of skin conditions, from acne scars and pigmentation to skin rejuvenation and vascular lesions [1, 2, 3]. Yet, despite their advancements, laser treatments remain susceptible to complications, particularly in patients with diverse skin types or complex skin conditions. The rise of artificial intelligence (AI) offers a powerful tool to enhance these treatments, providing unprecedented levels of precision, safety, and personalization [4]. AI is not just a buzzword in aesthetics; its application has been gaining real traction in the clinical setting [5]. AI is being integrated into diagnostic systems, laser devices, and treatment protocols, allowing dermatologists to fine‐tune their procedures for individualized results. By combining AI with laser technology, practitioners can potentially address long‐standing challenges, such as minimizing adverse effects, managing post‐treatment outcomes, and improving patient satisfaction [6]. The goal of this article is to explore the role of AI in enhancing laser‐based aesthetic treatments, examining its impact on skin analysis, treatment optimization, and post‐treatment monitoring. Ultimately, AI holds the promise of setting a new standard in aesthetic dermatology by reducing risks, maximizing outcomes, and delivering personalized care for a diverse patient population.

## 2 AI‐Driven Skin Analysis: A New Level of Customization

One of the most critical steps in ensuring successful laser treatments is an accurate understanding of a patient's skin characteristics. Traditional diagnostics rely heavily on manual inspection and the practitioner's experience. This approach, while effective, can sometimes lead to subjective results, especially for patients with more complex skin concerns or those with skin types that fall outside the typical parameters [2]. AI has changed this dynamic by offering precision diagnostic tools that can assess a patient's skin characteristics more thoroughly. AI algorithms can analyze thousands of data points, including melanin levels, vascular structures, pore size, and skin texture in a fraction of a second. The data collected through these algorithms provides practitioners with a more objective, comprehensive understanding of a patient's skin before proceeding with laser treatments [7]. For example, technologies such as AI‐powered dermatoscopes are now capable of conducting detailed, high‐resolution skin analyses to identify concerns like sun damage, fine lines, pigmentation, and textural irregularities with much greater accuracy than manual methods. Real‐time skin mapping is another leap forward in this context. Some platforms dynamically map the skin, highlighting regions where laser energy should be adjusted to avoid over‐treatment or under‐treatment. This technology minimizes risks in sensitive areas, such as the periorbital region or high‐pigmentation zones. By understanding these nuances, dermatologists can create treatment plans that are truly tailored to each patient, enhancing safety and efficacy [8]. These advanced diagnostics pave the way for highly customized treatment plans. Moreover, dermatologists can now create personalized treatment strategies that take into account the specific needs of the individual's skin, even before they come into contact with the laser procedure [9].

## 3 Optimizing Treatment Parameters With AI

One of the more revolutionary aspects of AI in aesthetic dermatology lies in its ability to optimize treatment parameters during laser procedures, moving beyond the current limitations of manual adjustments. In traditional laser therapy, practitioners rely on their experience and patient feedback to set parameters such as wavelength, energy level, and pulse duration. However, this approach carries a margin of error, especially for patients with darker skin types or complex conditions. AI could significantly reduce this uncertainty. By leveraging data from thousands of previous treatments, future deep learning algorithms could provide personalized recommendations, adjusting for factors like skin type, specific conditions, and treatment goals. These advanced algorithms could analyze aspects such as melanin levels, skin texture, and pore size to create a comprehensive skin profile, enabling more precise pre‐treatment planning. This is especially crucial for minimizing risks like post‐inflammatory hyperpigmentation (PIH) in patients with darker skin tones, where AI‐driven models might suggest specific wavelengths and energy levels to maximize safety and effectiveness [10].

A hypothetical scenario illustrates the potential impact: imagine an AI‐guided system that tailors laser settings for a patient with hyperpigmentation, making real‐time adjustments based on the skin's response to avoid adverse effects. Such a system, though not yet available, could offer unparalleled precision, adapting parameters instantly if signs of overheating or excessive trauma arise, thereby protecting the skin and enhancing treatment efficacy. While these AI algorithms are still in development, their imminent arrival signals a future where aesthetic laser treatments can be personalized with exceptional accuracy, improving both outcomes and safety for a diverse range of skin types.

## 4 Predictive Models for Post‐Treatment Results and Recovery

A significant advantage of AI in laser‐based treatments is its ability to predict post‐treatment outcomes. Historically, one of the challenges for patients undergoing laser treatments has been managing expectations. While before‐and‐after pictures help provide some context, individual outcomes vary, leaving a gap in patient understanding. AI addresses this through its predictive analytics capabilities [11]. Using extensive, transparent datasets from previous treatments, AI can predict not only the final aesthetic results but also the expected recovery timeline [12, 13]. AI‐powered models assess a patient's skin type, the specific laser settings used, and post‐treatment care to forecast outcomes. This enables practitioners to provide patients with a more realistic idea of what to expect, how their skin will respond, the likelihood of side effects, and how long the healing process will take. In some instances, AI systems can simulate what a patient's skin will look like after multiple treatment sessions. This type of visualization aids in consultations, allowing patients to make more informed decisions about their care. By showing expected results based on the specific parameters of the laser being used, it fosters trust between the patient and practitioner, improving patient satisfaction rates. AI can also identify patients at higher risk for complications, like PIH, allowing practitioners to take preventive measures or recommend specific pre‐care treatments. This personalized insight can significantly enhance patient satisfaction and safety. Moreover, AI can predict the risk of complications based on a combination of skin characteristics, laser settings, and patient history. For instance, a patient with a history of PIH might be flagged as high risk for hyperpigmentation post‐treatment. The practitioner can then make informed decisions to alter the treatment plan accordingly or recommend preventive pre‐care strategies [14].

## 5 AI‐Driven Post‐Treatment Monitoring

Post‐care is crucial in laser treatments, particularly in ensuring optimal results and preventing complications. AI systems are now being developed to assist in this phase as well. AI‐based apps can track a patient's recovery progress by analyzing daily photographs and feedback from the patient. These systems monitor the healing process in real‐time, looking for signs of abnormal recovery, such as prolonged redness, swelling, or blistering [15]. The AI system can then send alerts to the practitioner if there are any deviations from the expected healing trajectory, allowing for early intervention if necessary. Patients themselves can also receive tailored advice based on their recovery progress [16]. For example, if the AI detects that the skin is healing slower than expected, it might recommend adjustments to the patient's skincare routine, such as adding specific anti‐inflammatory creams or incorporating hydrating serums containing hyaluronic acid or suggesting antioxidants like vitamin C or niacinamide to support the skin's recovery and reduce inflammation. Another example, the AI system could advise on using gentle exfoliants to promote post treatment skin renewal. In addition to enhancing safety, AI‐driven post‐care recommendations can be tailored to each patient's recovery rate. For example, the AI system may suggest anti‐inflammatory creams or antioxidant serums if the recovery is slower than expected, or gentle exfoliants to promote skin renewal. This personalized post‐care support reinforces patient confidence and satisfaction, offering continuous care even after the treatment session. This real‐time post‐act monitoring enhances patient safety, reducing the likelihood of complications, and improves overall satisfaction by providing continuous, personalized care long after the treatment is completed.

## 6 Future Directions: The Next Frontier of AI and Laser Integration

As AI technology continues to evolve, its integration with laser treatments promises to reach new heights. Full integration of AI within laser devices could lead to autonomous adjustments of treatment parameters, achieving unparalleled precision. Future AI models might optimize combination treatments, like lasers with injectables or botulinum toxin, by analyzing patient data to recommend the optimal sequence and intensity for the best outcomes [18]. Wearable AI‐enhanced devices could also play a role, enabling real‐time monitoring of skin recovery through metrics like temperature and moisture levels, resulting in more accurate post‐care recommendations [17]. Virtual AI consultations, where patients receive customized treatment plans remotely, could further enhance patient engagement and improve treatment outcome*s*.

## 7 The Importance of Collaborative Efforts and Public Databases

The development of AI applications in aesthetic dermatology is a complex endeavor that requires collaborative efforts among researchers, clinicians, technology developers, and policymakers across various countries. Establishing a stakeholder taxonomy, a structured framework that identifies and categorizes all key players in AI development and application, ensures that each group's roles, contributions, and responsibilities are clearly defined. This framework is essential for fostering transparent collaboration, setting ethical standards, and aligning objectives across diverse groups. Furthermore, public databases are critical to facilitate AI advancements. Open‐access databases allow researchers to train algorithms on large, diverse datasets, improving AI's ability to generalize across different skin types, conditions, and demographics. By pooling data resources, stakeholders can accelerate AI's development, refine its accuracy, and democratize its benefits, leading to safer, more effective applications for a broader range of patients. Thus, building and maintaining these shared databases is a crucial step toward maximizing the potential of AI in aesthetic and clinical dermatology.

## 8 Ethical and Practical Challenges of AI in Aesthetic Dermatology

Although AI offers groundbreaking advancements in aesthetic dermatology, it also presents ethical and practical challenges that must be carefully addressed. A key concern is algorithmic bias, where AI trained on non‐diverse datasets may produce less effective or even unsafe results for underrepresented groups, such as patients with darker skin tones. This necessitates inclusive data collection and performance audits to ensure fair and accurate recommendations for all demographics. Additionally, data privacy and security are crucial, as AI systems require extensive personal data to function effectively [12, 19]. Compliance with regulations, secure data storage, and transparent consent practices are essential to protect patient confidentiality and maintain trust. Therefore, AI‐driven healthcare must prioritize these protections, particularly given the sensitivity of medical data. Furthermore, standardized regulatory oversight is needed to validate AI's accuracy, safety, and reliability [20]. Currently, the lack of universal guidelines for AI tools can cause variations in quality. To address this, standardized testing and regulatory frameworks are needed to ensure AI applications meet safety and effectiveness standards before widespread use [21]. Creating open‐access databases can also help by providing diverse data for training, which improves the accuracy and relevance of AI tools. However, these databases must carefully protect patient privacy and confidentiality. Reducing healthcare disparities through accessible AI is crucial. If AI tools are designed for a narrow range of users, they risk increasing inequalities. Making AI affordable, effective, and widely available can help more people benefit from these technologies. By committing to ethical standards, like addressing bias, ensuring data privacy, promoting transparency, and supporting equitable access, AI can more effectively advance aesthetic dermatology safely and inclusively.

## 9 Conclusion

The integration of AI and laser treatments is set to transform aesthetic dermatology by enhancing precision, safety, and personalization. AI‐driven diagnostics, real‐time treatment optimization, and predictive recovery models offer tailored solutions, particularly benefiting patients with darker or more complex skin conditions. As AI reduces the margin for error, dermatologists who adopt these tools will provide more advanced, effective, and personalized care. However, clear guidelines are crucial to ensure safety, data privacy, and rigorous validation, while fair frameworks are needed to ensure accessibility and equity. However, establishing clear guidelines and ethical standards is critical to ensuring equitable access and rigorous validation. By combining these elements, AI can elevate clinical outcomes and the patient experience, setting a new standard in aesthetic dermatology.

## Ethics Statement

The author has nothing to report.

## Conflicts of Interest

The author declares no conflicts of interest.

## Data Availability

The data that support the findings of this study are available on references' part.
